# Supplementary material for: Synthesis of cBN-hBN-SiCw Nanocomposite with Superior Hardness, Strength, and Toughness
Source: Nanomaterials (Basel). 2022 Dec 22;13(1):37. doi: 10.3390/nano13010037 (PMC9823861; doi:10.3390/nano13010037)
Supplement: Supplementary file 1 [file nanomaterials-13-00037-s001.zip › nanomaterials-2049461-supplementary.pdf]

nanomaterials

MDPI

Supplementary Materials

Synthesis of cBN-hBN-SiC_w Nanocomposite with Superior Hardness, Strength, and Toughness

Lei Sun [1,†], Yitong Zou [1,†], Mengdong Ma [1,2,*], Guangqian Li [1], Xiaoyu Wang [1], Xiang Zhang [1], Zewen Zhuge [1], Bing Liu [1], Yingju Wu [1], Baozhong Li [1] and Zhisheng Zhao [1,*]

[1] Center for High Pressure Science (CHiPS), State Key Laboratory of Metastable Materials Science and Technology, Yanshan University, Qinhuangdao 066004, China

[2] Macao Institute of Materials Science and Engineering, Macau University of Science and Technology, Taipa, Macao 999078, China

* Correspondence: zzhao@ysu.edu.cn (Z.Z.); mamengdong1991@163.com (M.M.)

† These authors contributed equally to this work.

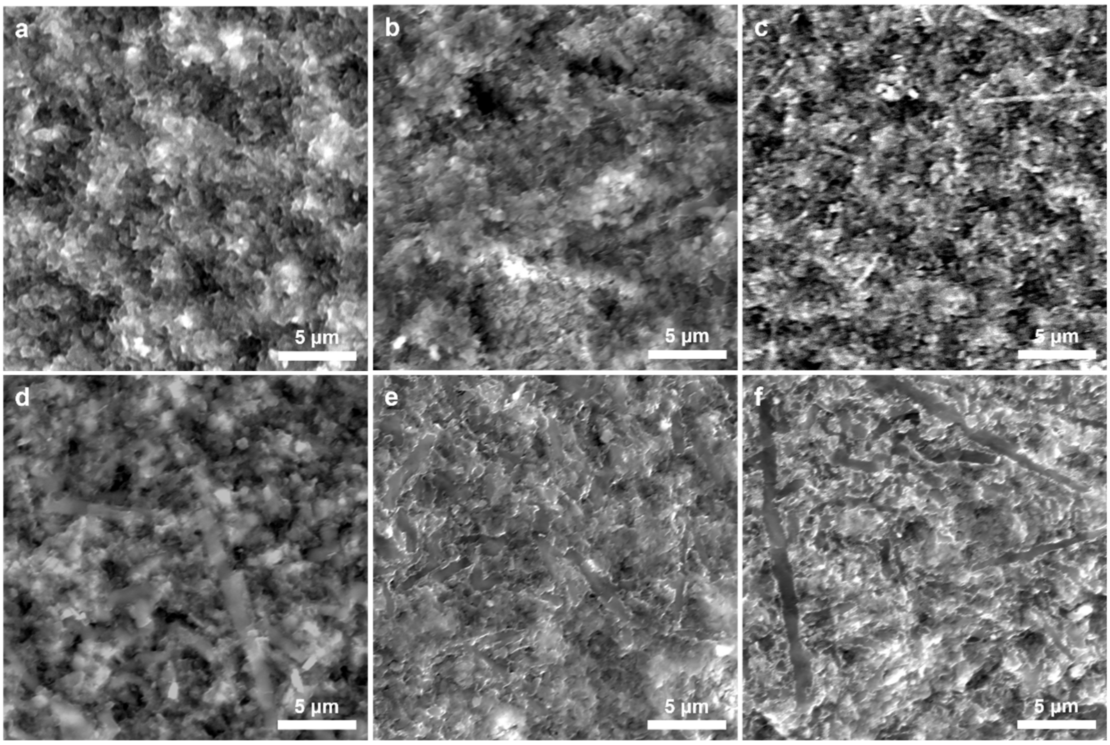

Figure S1. SEM images of fracture surfaces for the nanocomposites with **(a)** 0 wt.% SiC_w, **(b)** 5 wt.% SiC_w, **(c)** 10 wt.% SiC_w, **(d)** 15 wt.% SiC_w, **(e)** 20 wt.% SiC_w, and **(f)** 30 wt.% SiC_w.

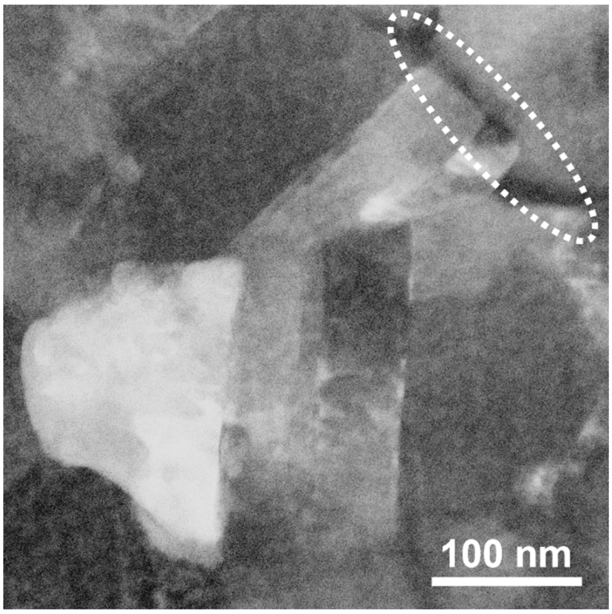

Figure S2. STEM image of the nanocomposite with 25 wt.% SiC_w. The hBN is marked by the white circles.

Table S1. Interplanar spacing of cBN, SiC, and hBN identified by TEM, XRD, and PDF card.

Phase	Crystal plane	HRTEM	XRD	PDF card
cBN	(111)	2.09 Å	2.09 Å	2.087 Å
SiC	(111)	2.52 Å	2.52 Å	2.520 Å
hBN	(0002)	3.11 Å	3.11 Å	3.328 Å
